# The Development and Analysis of a Preliminary Electrodialysis Process for the Purification of Complex Lithium Solutions for the Production of Li_2_CO_3_ and LiOH

**DOI:** 10.3390/membranes15020050

**Published:** 2025-02-05

**Authors:** Alonso González, Geovanna Choque, Mario Grágeda, Svetlana Ushak

**Affiliations:** 1 Center for Advanced Study of Lithium and Industrial Minerals (CELiMIN), Universidad de Antofagasta, Campus Coloso, Av. Universidad de Antofagasta, Antofagasta 02800, Chile; alonso.gonzalez@uantof.cl (A.G.); geovanna.choque.guisbert@ua.cl (G.C.); svetlana.ushak@uantof.cl (S.U.); 2 Departamento de Ingeniería Química y Procesos de Minerales, Universidad de Antofagasta, Av. Universidad de Antofagasta, Antofagasta 02800, Chile

**Keywords:** LiCl solution, membrane electrolysis, electrodialysis, Mg removal, Ca removal

## Abstract

Direct lithium extraction (DLE) is emerging as a promising alternative to brine extraction although it requires further processing to obtain high-quality products suitable for various applications. This study focused on developing a process to concentrate and purify complex LiCl solutions obtained through direct lithium extraction (DLE). Two different chemical compositions of complex LiCl solutions were used, dividing the study into three stages. In the first part, lithium was concentrated to 1% by mass by evaporation. In the second, electrodialysis was used to alkalinize the LiCl solution and remove magnesium and calcium impurities under different current densities. The best results obtained were magnesium and calcium removals of 99.8% and 98.0%, respectively, and lithium recoveries of 99% and 96%. In the third stage, the selectivity of two different commercial cationic membranes (Nafion 117 and Neosepta CMS) was evaluated to separate Li^+^, K^+^, and Na^+^ cations under different current densities and volumetric flow rates. The Neosepta CMS membrane demonstrated higher lithium recovery. This study evaluated the quality of the purified lithium-rich solution and its potential use both in the production of Li_2_CO_3_ as well as in the electrochemical production of LiOH.

## 1. Introduction

The conventional process of obtaining lithium compounds consists of concentrating the brine in evaporation ponds and then using chemical agents to produce Li_2_CO_3_ and LiOH [1,2,3,4]. However, this method presents challenges, such as significant water loss through evaporation and the generation of solid waste from chemical reactions. Current production in Chile evaporates more than one hundred cubic meters of brine per ton of Lithium Carbonate Equivalent (LCE), generating a high water and carbon footprint, affecting aquifers and local communities [5]. The conventional process of lithium extraction and concentration faces technical, environmental, and operational challenges. From a technical perspective, the evaporation concentration process takes between 12 and 18 months to achieve a lithium concentration of 4–6% by weight. This requires extensive facilities and a global recovery efficiency ranging from 43% to 49%. These figures are impacted by unwanted lithium losses in the form of lithium carnallite. Additionally, if sulfate is present as an impurity, there can be losses of lithium sulfate in the precipitation of potassium carnallite [6].

In 2023, the Chilean government announced a restructuring of the lithium industry. One of the measures was that private companies must adopt processes based on direct lithium extraction (DLE) that minimize environmental impact and maximize lithium recovery [7].

DLE involves various technologies such as solvent extraction and electrochemical, adsorption, and ion exchange resin methods [8]. Among the processes of adsorption and ion exchange, sorbents such as aluminum-based sorbents, LMOs, LTO, and HMO exhibit a high affinity for Li^+^. Specifically, the HMO material shows a high selectivity for Li+ due to the small pore window in the spinel structure of MnO_2_ in ion exchange processes [9]. These materials are arranged in columns, where lithium is extracted by depleting the brine, which has the potential to be reinjected back into the salar. Subsequently, the resins loaded with Li are treated with acidic solutions to desorb the Li^+^ cations, producing a lithium-rich solution [9,10,11]. This study used LiCl solutions obtained after a DLE desorption process based on ion-exchange as the initial feed. While these solutions are rich in lithium, they are complex because they carry impurities of Na, K, Mg, and Ca. Additionally, the complex solution has an acidic pH due to the sorbent regeneration process and presents a lithium concentration of less than 0.1% by weight. This concentration is low since around 1% lithium is required for direct use in the production of Li_2_CO_3_ by carbonation [12]. Similarly, a high concentration is also required for use in the production of LiOH through electromembrane processes [13,14].

DLE processes produce lithium-enriched solutions that still carry impurities, requiring further refining [15,16,17].

Direct lithium extraction processes, mainly those based on adsorption and ion exchange, require a desorption stage with acidic solutions. Depending on the type of material used as a sorbent, in the case of Lithium Ion Sieves (LISs), the Li+ adsorption capacity ranges from 9.63 to 39.5 mg/g of the sorbent, with the main impurities being Na^+^ (0–47.38), K^+^ (0.07–9.63 mg/g), Mg^2+^ (0–71.87 mg/g), and Ca^2+^ (0–2.11 mg/g) [18]. After desorption, these impurities are carried into the resulting product solution. A notable case study was that of Köbel et al. [19], using adsorption and ion exchange to treat geothermal brines. After sixteen extraction cycles, the chemical composition showed a significant Na^+^ content (213 mg/L), followed by Li^+^ and Ca^2+^ (133 mg/L), and then K^+^ and Mg^2+^ with contents lower than 20 and 10 mg/L, respectively. The carryover of these impurities can be explained by the designs of columns or non-optimized washing processes.

EDL technologies mostly require pre-treatment to reduce impurities or modify the pH, thus improving process efficiency [8]. Technologies used for impurity removal include nanofiltration, which allows for lithium concentration while separating 99% of divalent ions like Ca^2+^ and Mg^2+^ and 40–50% of monovalent ions like Na^+^ and K^+^ [20].

Additionally, EDL processes require post-treatment to concentrate, refine, and convert lithium into products like lithium salts [8]. In refining, solvent extraction, ion exchange, and precipitation are commonly used processes [21]. Membrane and ion exchange processes show good results in removing impurities like Na, K, Mg, and Ca [22]. The main challenges in the post-treatment of solutions from DLE include high energy consumption and difficulties in achieving high purity in final lithium products [8].

Electrodialysis combines the use of ion exchange membranes and the possibility of using renewable energies due to its electrochemical nature. Compared to conventional/membrane-based adsorption, electrodialysis practically does not consume chemical reagents, has faster kinetics, and is versatile, allowing for cell configuration adaptation for various applications [23]. Furthermore, since it can be powered by renewable energy sources, it becomes a promising alternative for its use in purification and impurity removal processes.

Therefore, our paper focuses on developing and evaluating a method for impurity separation in the solution resulting from the DLE process. This approach aims to connect direct lithium extraction (DLE) processes from brines with lithium compound production processes.

Several studies were focused on improving purification processes removing major impurities such as Na, K, Ca, and Mg. In the removal of divalent ions, Tran et al. [24] employed a chemical precipitation method to purify a LiCl brine sourced from the Uyuni salt flat, with Mg/Li and Ca/Li ratios of 21 and 0.7, respectively. In this method, Ca and Mg compounds were precipitated by adding oxalic acid under conditions of an oxalate/Ca molar ratio of 6.82 at a pH below 1 and an oxalate/Mg ratio of 1.62 within a pH range of 3 to 5.5. The results indicated a final composition of 0.003% Ca, 0.83% Mg, 67.6% Na, 10.6% K, and 0.41% Li, achieving an 80% removal of Ca and a 95% removal of Mg, with Li losses of 35%. On the other hand, Firdiyono et al. [25] also investigated the addition of oxalic acid, and their results showed a reduction in Li losses by working at pH 4. By adjusting the oxalic acid ratio, it was possible removal of 99% of Mg in the first stage of the process. Additionally, the study by Grágeda et al. [26] developed a chemical precipitation process to remove the main impurities of calcium, magnesium, and sulfate from a concentrated lithium brine with Mg/Li, Ca/Li, and SO_4_/Li ratios of 0.22, 0.01, and 0.005, respectively. The removal efficiencies achieved for calcium, magnesium, and sulfate were 98.93%, 99.93%, and 97.14%, respectively. Subsequently, the use of ion exchange resins reduced the concentrations of Ca and Mg to values below 1 ppm. This process achieves very low concentrations of calcium and magnesium ions, where the allowed concentration ranges for calcium are between 1 and 3 ppm, for magnesium between 0 and 10 ppm, and for sodium between 20 and 30 ppm [26].

Chemical precipitation processes have proven to be effective and are therefore used at the industrial level. However, the challenge over the years has been to develop more sustainable processes that do not generate waste or cause significant water losses, such as electrochemical processes. For example, Zhao et al. [27] simulated a brine with the Li and Mg composition from the West Taijnar Salt Lake (China), with Mg/Li and Ca/Li ratios of 60 and 1.4, respectively. They employed an electrochemical separation method to extract and concentrate solutions containing Mg from Li. They utilized the electrochemical difference and the reversibility of the LiFePO_4_/FePO_4_ electrode structures [28]. This method allowed the Mg/Li ratio to be reduced from 60 to 0.45 due to the high selectivity of LiFePO_4_ for Li. The method enables the extraction of 25% of lithium in a single operation in the presence of Mg, even with Mg/Li ratios as high as 110. Similarly, Xu et al. [29] used an electrochemical intercalation and deintercalation process with a brine that had a Mg/Li ratio of 58.5, which was reduced to 0.93 in the anolyte. Following this process, nanofiltration, reverse osmosis, evaporation, and carbonation were used by adding Na_2_CO_3_ to produce lithium carbonate.

Ji et al. [30] used an electrodialysis (ED) system with a monovalent selective ion exchange membrane. This technology demonstrated higher separation efficiency for initial Mg/Li ratios of 60, and relationships of Mg/Li ranging from 5 to 92 were also studied. Over a period of 2 h, the Mg/Li ratio was reduced to 7, with a Li recovery of 72.46%. The effects of applied voltage, linear flow velocity, Mg/Li ratio, and pH on lithium separation were analyzed. This method shows promise for lithium recovery from brines with high Mg/Li ratios. Chen et al. [31] studied the selective electrodialysis (S-ED) method to examine the influence of coexisting cations in the brines (Na^+^ and K^+^) and their negative effect on lithium ion migration, using monovalent selective ion exchange membranes. In the initial phase, a NaCl solution was added to the concentration compartment, Na_2_SO_4_ to the electrolyte compartment, and 0.035% Li brine to the desalination compartment, with Na/Li and K/Li ratios of 1, 3, 5, 10, 15, 20, and 30. With Na/Li ratios between 1 and 3, Li recovery increased linearly during the first 60 min, after which recovery slowed. However, with Na/Li ratios between 20 and 30, Li recovery remained at a lower level, and the order of the negative influence of the coexisting cations on lithium migration was inversely related to their hydrated radius: K^+^ > Na^+^ > Ca^2+^ > Mg^2+^.

Díaz Nieto et al. [32] propose electrodialysis with membranes as an alternative for the removal of divalent cations from brines sourced from the Salar del Hombre Muerto in northern Argentina. In the cathodic compartment, natural brine (initial composition: 0.309% Mg and 0.068% Ca) is added, where Mg(OH)_2_ and Ca(OH)_2_ precipitate. In the anodic compartment, HCl is used, and a phosphate buffer solution is added, adjusted with NaOH to maintain a pH of 10, ensuring that the Cl_2_ formed is disproportionated into Cl and ClO^−^. For studies with Li concentrations of 0.13%, 0.059%, and 0.127 ppm, with Mg/Li ratios of 2.4, 4.6, and 6.9, respectively, the lowest energy consumption was 62 kWh/m^3^ of brine for the complete removal of both cations to concentrations lower than 2 ppm. Similarly, Pan et al. [33] proposed an electrochemical method to separate and extract Mg and Li from brines. In this method, the brine with an Mg/Li ratio of 98.23 enters the anodic compartment and the Mg^2+^ cations migrate to the cathodic compartment where Mg(OH)_2_ precipitates. Part of the lithium migrates with magnesium, enriching the catholyte, while the rest of the Li remains in the anolyte, forming a lithium-rich liquid with a low Mg/Li ratio. Through multistage electrolysis, the Mg/Li ratio can be reduced from 98 to 0.17, achieving final concentrations of 0.026 g/L of Mg and 0.15 g/L of Li. This method allows for an excellent reduction of the Mg/Li ratio from 98.23 to 0.17.

Most DLE processes face challenges when attempting to obtain impurity-free LiCl, necessitating the development of secondary purification processes to meet battery-grade standards [13,16]. The aim of this study was to evaluate the concentration and purification of a complex LiCl solution obtained through direct lithium extraction (DLE) to prepare it for the subsequent production of Li_2_CO_3_ via carbonation or LiOH via membrane electrolysis; both routes were analyzed.

The existing challenges in carrying out this task were as follows. The lithium solution obtained in direct lithium extraction (DLE) processes has a low lithium concentration (less than 0.1%), which requires it to be concentrated to 1% by mass before it can be used in a carbonation or membrane electrolysis process. Depending on the initial brine, the lithium solution may contain significant impurities of Na, K, Mg, and Ca. For carbonation with Na_2_CO_3_, it is essential to remove divalent ions, as they may precipitate alongside Li_2_CO_3_, while for membrane electrolysis, it is important to eliminate as many non-lithium cations as possible. Additionally, the final solution obtained from DLE has a highly acidic pH due to the lithium being desorbed in acidic solutions (such as HCl, H_2_SO_4_, among others), which complicates the removal of Ca^2+^ and Mg^2+^ impurities in ion-exchange-resin-based processes. Therefore, the working solution needs to be alkalinized.

Our work preliminarily addresses these challenges by using evaporation to concentrate the lithium solution and electrodialysis and alkalinization to adjust the pH and facilitate the removal of divalent impurities. And finally, the effect of monovalent ions to obtain LiOH by membrane electrolysis is evaluated, defining the scope and opportunities for improvement.

## 2. Materials and Methods

The methodology focused on evaluating a pre-treatment for complex LiCl solutions obtained from DLE technologies, primarily in those acidic solutions obtained in adsorption and ion exchange processes. Two solutions with different chemical compositions (Table 1) were simulated, which mainly differed in salinity, primarily due to sodium content, and in the ratios of Na/Li, K/Li, Mg/Li, and Ca/Li.

To prepare the lithium-rich solutions, HCl 37%, LiCl, KCl, NaCl, MgCl_2_·6H_2_O, and CaCl_2_·2H_2_O reagents from the Merck Millipore line (Billerica, MA, USA) with a minimum purity of 99% were used. Solution L1 simulated a diluted lithium-rich chemical composition while solution P1 simulated one with a higher content of impurities.

The process was developed and evaluated in three stages, described below:

- First Stage: The objective was to concentrate the lithium-rich solution obtained from DLE to 1% Li by weight. This was achieved by an evaporation concentration process where the brines PC1 and LC1 were obtained, referred to as the initial solutions P1 and L1 after concentration.
- Second Stage: A membrane electrodialysis process was used to alkalinize the acid solution, studying the removal of divalent cations (Mg^2^⁺ and Ca^2^⁺) by precipitation of Mg(OH)_2_ and Ca(OH)_2_. As a result, the brines PC2 and LC2 were obtained.
- Third Stage: The best result from the second stage was further worked with, evaluating the selectivity of the Na⁺ and K⁺ impurities still present alongside the Li⁺ ion. A commercial cationic membrane Nafion 117 (DuPont Co., Wilmington, DE, USA) and a monovalent selective cationic membrane Neosepta CMS (ASTOM Co., Tokyo, Japan) were used in a membrane electrolytic reactor prototype. The LiCl feed solution (PC2 and LC2) would serve as the anolyte, and the resulting LiOH solution would serve as the catholyte (PC3 and LC3), allowing for the evaluation of Li recovery and specific energy consumption.

### 2.1. Concentration by Evaporation

Evaporation is the method used to concentrate the LiCl solution after DLE (Table 1). The objective is to increase the concentration to 1% Li, considering the presence of impurities such as Na, K, Mg, and Ca. The equipment used for the experimental tests was a Rotavapor R-210 (Büchi Labortechnik AG, Flawil, Switzerland). The vacuum generated at 72 mbar in the rotary flask lowered the boiling point of water to approximately 40 °C. After evaporation, the brines LC1 and PC1 were obtained.

### 2.2. Electrochemical Alkalinization Process

In this stage, the objective is to remove the divalent cations in the LiCl solutions, such as Mg and Ca. To achieve this, the electrodialysis process is proposed. The effect of concentration was analyzed by comparing the solution P1 with the brine PC1 while the effect of chemical composition was evaluated by comparing the brine PC1 with LC1. It is important to highlight that solution P1 was only considered for comparative purposes as its low concentration made it unsuitable for the efficient production of Li_2_CO_3_.

**Figure 1:**
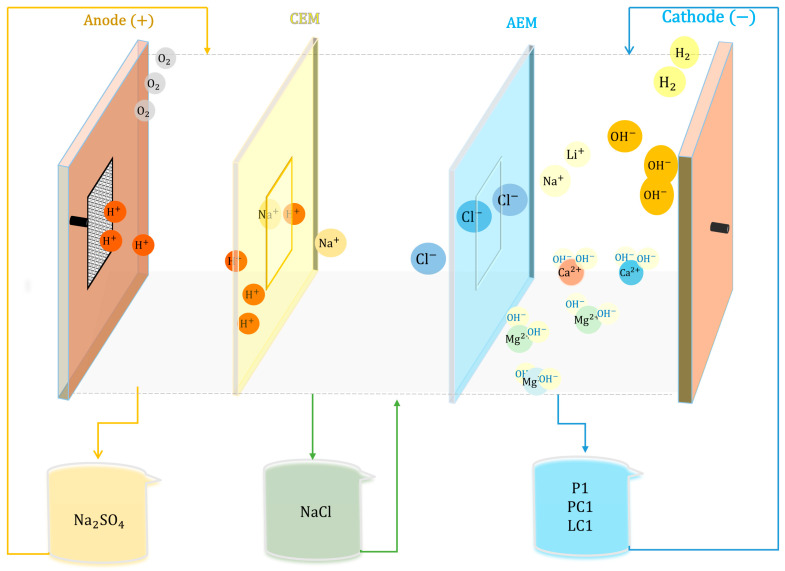
Representative schematic of the electrodialysis system with three compartments. CEM: cation exchange membrane; AEM: anion exchange membrane.

The electrochemical alkalinization process was conducted using a three-compartment electrolyzer prototype. A Neosepta AMX anionic membrane and a Neosepta CMX cationic membrane (ASTOM Co., Tokyo, Japan) were used according to Figure 1. The flow rate was adjusted to 1.67 cm/s in each compartment using three peristaltic pumps (Watson-Marlow 520SN/R2, Falmouth, UK). To provide a direct current source, a GW Instek GPR-1810HD rectifier was used (New Taipei, Taiwan). The process was performed at room temperature.

In the anolyte compartment, 500 g of 1 M Na_2_SO_4_ was introduced so that through water oxidation, protons (H^+^) were generated, and H_2_SO_4_ was formed (Equation (1)). The free Na⁺ ions would migrate towards the intermediate compartment through the cationic membrane Neosepta CMX.

(1) $$2H_{2}O\to O_{2}+4H^{+}+4e^{-}$$

In the intermediate compartment, an initial solution of 500 g of 0.2 M NaCl was used, which was concentrated through the migration of Cl^−^ ions from the catholyte via the Neosepta AMX membrane and Na⁺ ions from the anolyte. Both membranes had an area of 30 cm^2^.

In the catholyte, 500 g of the initial LiCl feed, containing the main impurities of Ca^2^⁺, Mg^2^⁺, Na⁺, and K⁺, was introduced. The initial LiCl feed had an acidic pH, so at the cathode, through the reduction of water, OH⁻ ions were generated (Equation (2)), contributing to the increase in pH. This caused Mg^2^⁺ and Ca^2^⁺ ions to precipitate as Mg(OH)_2_ and Ca(OH)_2_ due to their low solubility, thereby removing these impurities from the LiCl solution, as shown in Equations (3) and (4).

(2) $$2H_{2}O+2e^{-}\to H_{2}+2OH^{-}$$

(3) $$Mg^{2+}+2OH^{-}\to Mg{\left(OH\right)}_{2}$$

(4) $${Ca}^{2+}+2OH^{-}\to Ca{\left(OH\right)}_{2}$$

With this configuration, the removal of Mg and Ca from the LiCl solution was achieved without the need to add any precipitating agents. Sulfuric acid was obtained as a byproduct, along with H_2_ and O_2_ gases, which do not have negative environmental impacts. It is important to note that this setup avoids the formation of Cl_2_.

The experimental design for these tests is detailed in Table 2, with the current density as the controllable factor and the dependent responses being the operating time and the change in pH. The effect of temperature is not considered because an increase would result in increased solubility, which is undesirable, and it would also represent an additional energy cost.

To identify the tests under different operating conditions, they are labeled according to the initial LiCl solution (PC1, LC1, and L1) and the experiment number in Table 2. For example, for the brine PC1, the tests are defined as PC11, PC12, PC13, PC14, and PC15, where the last digit corresponds to the experiment number. As a result of the alkalinization, the best results will be labeled as brines PC2 and LC2, which have potential use in carbonation with Na_2_CO_3_ to obtain Li_2_CO_3_.

The specific energy consumption per initial lithium concentration treated (SEC) is calculated using Equation (5).

(5) $$SEC=\int_{0}^{t}\frac{E\cdot I}{m_{Li}}dt$$

Here, E(V) is the voltage drop across the cell, I is the applied current, and $m_{Li}$_+_ is the mass of lithium recovered.

The removal percentage indicates the effectiveness of the process in removing Mg and Ca impurities from the feed stream and is calculated using Equation (6):

(6) $$\%Removal=\left(\frac{C_{o}M_{o}-C_{f}M_{f}}{C_{o}M_{o}}\right)\times 100\%$$

Here, $C_{o}$ and $C_{f}$ are the initial and final concentrations of the substance and $M_{o}$ and $M_{f}$ correspond to the initial and final mass of the catholyte.

The lithium recovery percentage is calculated using Equation (7).

(7) $$\%Removal=\frac{C_{Lif}M_{f}}{C_{Lio}M_{o}}\times 100\%$$

Here, $C_{Lio}$ and $C_{Lif}$ are the initial and final lithium concentrations in the catholyte while $M_{o}$ and $M_{f}$ are the initial and final mass of the catholyte.

### 2.3. Membrane Electrolysis Process

The objective of this stage was to study how the impurities of Na and K that accompanied Li in the PC2 and LC2 solutions affected the membrane electrolysis process to produce a LiOH solution as an alternative to the Li_2_CO_3_ production process. This will be evaluated by the migration rates of the monovalent cations and the selectivity of these cations across the Nafion 117 (DuPont Co., Wilmington, DE, USA) and Neosepta CMS (ASTOM Co., Tokyo, Japan) membranes. Finally, the results will be evaluated in the cathodic compartment, and the influence of Na and K impurities on lithium recovery will be analyzed. These brines are considered free of Mg and Ca cations, with only differences in the Na/Li and K/Li ratios.

**Figure 2:**
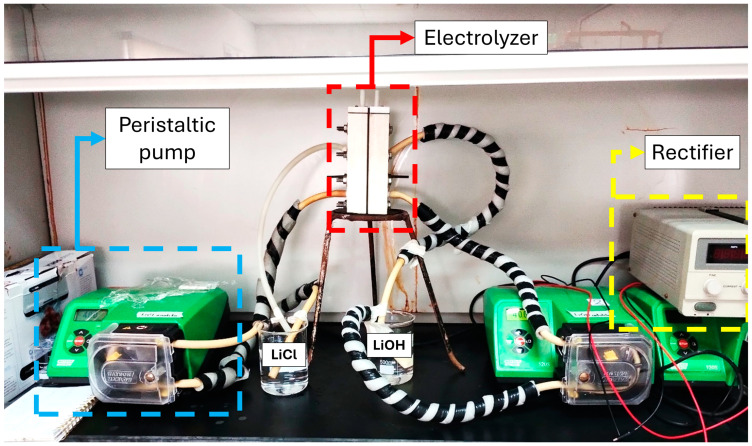
Experimental setup of the membrane electrolysis process—Stage 3.

The electrolyzer used in this study (Figure 2) was a prototype piston flow reactor made of Teflon with a customized design, featuring two compartments separated by a cationic membrane.

In the experimental development, the factors to be controlled were the current density and the flow rate for each type of membrane, with a constant operating time of 1 h. Using a 2 k design with a midpoint, a total of 5 tests were conducted for each type of membrane, as shown in Table 3. Current density levels ranged from 500 to 1500 A/m^2^, and RPM ranges were between 40 and 170, equivalent to linear flow velocities between 1 and 4.5 cm/s, respectively.

**Figure 3:**
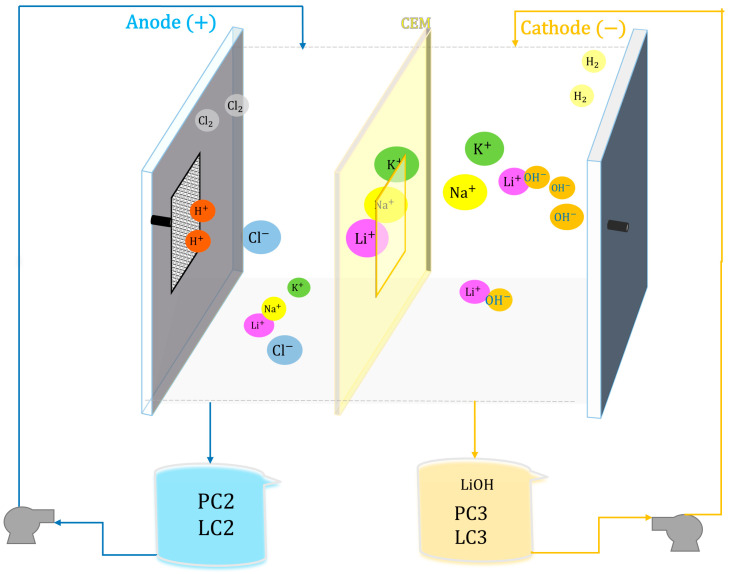
Experimental scheme of membrane electrolysis—Stage 3.

Table 3 presents the test setup for the PC2 solution; the conditions that yielded the best results will be replicated with the LC2 solution for comparative purposes. Figure 3 shows the schematic of the electrolysis process, where the cathodic compartment contains 0.5% by mass LiOH solution and the anodic compartment contains the LiCl solution (PC2 or LC2). The cationic membrane allows the passage of positive cations (Li^+^, Na^+^, and K^+^) from the anolyte to the catholyte. At the anode, H^+^ protons, O_2_ gas, and Cl_2_ gas are generated, while at the cathode, OH⁻ is produced by the reduction of water. In this way, monovalent cations migrate to the cathodic compartment to form LiOH, NaOH, and KOH. The membrane has an area of 30 cm^2^ and the surface area of the electrodes is 31.5 cm^2^.

The recovery ratio of Li ($R_{Li}$) is calculated by Equation (8):

(8) $$R_{Li}\left(\%\right)=\frac{M_{ct}\left(C_{ct}-C_{c0}\right)}{M_{a0}\cdot C_{a0}}\times 100\%$$

Here, $C_{c0}$ and $C_{ct}$ are the initial and final concentrations of lithium ions in the cathodic compartment, respectively. $M_{ct}$ is the mass of the LiOH solution concentrated at time *t*. $M_{a0}$ and $C_{a0}$ are the mass of the solution and the concentration of lithium ions in the LiCl solution in the anodic compartment, respectively.

#### Statistical Analysis

In stage 3 of the membrane electrolysis process, the main effects and interactions of the factors current density (A), flow rate (B), and membrane type (C) in relation to the Na/Li and K/Li ratios were studied. The data were analyzed using linear regression and analysis of variance (ANOVA). The experimental design was orthogonal, as confirmed by the variance inflation factors (VIF), all of were equal to 1.0, indicating that the effects were independent. The standard error (SE) was calculated according to Equation (9) using the standard deviation (s) and the sample size (n):

(9) $$SE=\frac{s}{\sqrt{n}}$$

The analyses were performed using Statgraphics version 19.4.04 (Statgraphics Technologies, Inc. The Plains, VA, USA) with a significance level of 5% (α = 0.05).

## 3. Results

### 3.1. Concentration of Solutions

Through evaporation, concentrated brines PC1 and LC1 were obtained, with their chemical compositions specified in Table 4. The final pH of both solutions was 1.2. It was observed that it was possible to concentrate solutions P1 and L1 to more than 1% lithium by mass, with brine PC1 being close to saturation due to its high sodium content. In addition, the PC1 brine contained more magnesium while the LC1 brine contained more calcium. The specific energy consumption was determined by considering the mass of water that was evaporated, resulting in 0.55 and 0.60 kWh per kilogram of solution for brines PC1 and LC1, equivalent to 52.38 and 52.45 kWh per kilogram of lithium, respectively.

### 3.2. Electrodialysis Process for Mg and Ca Removal

#### 3.2.1. pH Evolution in Electrodialysis Process

In the electrodialysis cell, the pH changed in each compartment. In the anodic compartment, the dissociation of sodium sulfate caused Na⁺ ions to migrate to the intermediate compartment, acidifying the anolyte through the formation of sulfuric acid. On the other hand, in the cathodic compartment, the formation of OH⁻ raised the pH of the catholyte solution (LiCl solution), causing the precipitation of Mg^2^⁺ and Ca^2^⁺. In the middle compartment, NaCl was concentrated, and due to the migration of H⁺ from the anodic compartment, the solution became acidified, resulting in a NaCl solution with a final pH of 3.

**Figure 4:**
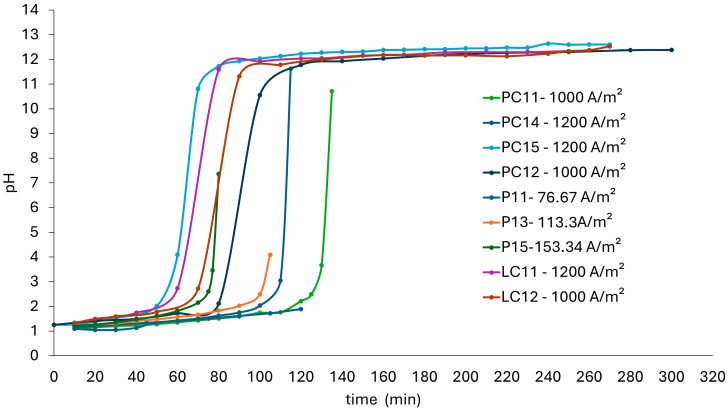
Variation in pH in the catholyte and specific energy consumption according to current density and operation time.

The precipitates in the cathodic compartment increased over time based on the solubility products, where Mg(OH)_2_ precipitated before Ca(OH)_2_. The charge circulating in the system controlled the pH of the solution and the reaction time. Since the current remained constant, the pH graph in the catholyte versus the elapsed time can be observed in Figure 4.

In the direct solutions from the EDL (P11, P13, and P15), the initial pH was 1.2 and gradually increased to approximately pH 2.5, after which there was a sharp rise in pH, with the magnitude depending on the applied current density. This was attributed to higher current densities promoting greater OH⁻ generation, which reacted with Mg^2^⁺ ions to form Mg(OH)_2_. A pH above 4 is considered suitable for divalent cation removal processes using ion exchange resins, which represents a potential process alternative.

The operation time increased when lower current density values were used, indicating that reaching a higher pH requires more time for electrolyte recirculation, which increases the energy required for pumping.

Regarding the concentrated solutions (PC1), tests of PC11 and PC14 were alkalized to a pH close to 11 while tests of PC12 and PC15 were extended over time to observe the pH increase. In this figure, three defined regions are observed: first, a region of slow pH increase from 1.2 to 2.1, followed by a sharp pH change to a range of 10.2 to 11.6, and afterward, a region with little pH variation between 11 and 12.5. Upon reaching a pH between 10 and 11, it stabilized because Ca^2^⁺ started to consume the OH⁻ produced, forming Ca(OH)_2_. A further increase in pH was expected once Ca^2^⁺ was depleted, but this did not occur even after 300 min of operation, resulting in higher energy consumption. This was because at lower Ca concentrations, the molecular interaction is reduced and the removal process is slower. This can be attributed to the characteristics of the Mg(OH)_2_ and Ca(OH)_2_ formed, which reduced the interaction between the remaining Ca^2^⁺ and the OH⁻ ions generated in the cathodic half-reaction.

At current densities of 1000 and 1200 A/m^2^, there was a 20 min difference in the final precipitation times for Mg and Ca in each sample. For the PC1 brine, the specific energy consumption was between 16 and 30% lower at 1000 A/m^2^, although its operation time was 11–17% longer than at 1200 A/m^2^. In contrast, when using the LC1 brine, energy consumption was 19% lower at 1000 A/m^2^, and the operation time was 4% longer. Precipitating Mg takes an average time of 80 min while removing Ca extended the time to 270 min (4.5 h). The results indicate that it is preferable to operate at a current density of 1000 A/m^2^ as it provides a high removal of Mg and Ca along with lower specific energy consumption.

#### 3.2.2. Li Recovery and Specific Energy Consumption

**Figure 5:**
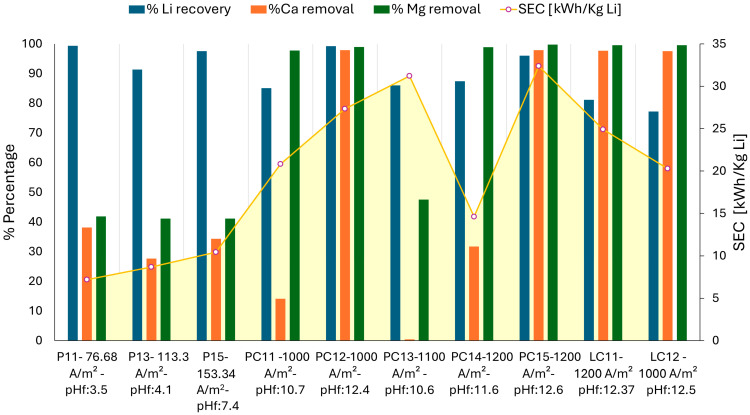
Results of lithium recovery, impurity removal, and specific energy consumption.

In Figure 5, the decreases in Mg and Ca for the different configurations are observed. Better results were obtained in concentrated solutions (PC1 and LC1) than in diluted solutions (P1). This was because the higher concentration of salts made more Mg and Ca ions available to react with the OH^−^ ions.

Magnesium hydroxide (Mg(OH)_2_) precipitated first due to its lower solubility compared to calcium hydroxide (Ca(OH)_2_). On the other hand, the formation of LiOH precipitates did not occur because LiOH has a higher solubility (greater than 10 g/100 g solution at room temperature) [34], with the Li ion remaining in the aqueous phase. In the experiments involving solution P1, the removal of Mg and Ca was consistent across all tests, demonstrating independence from the applied current density. The removal efficiencies for magnesium and calcium were similar in tests using solution P1, likely due to the increased production of OH⁻ at higher currents (153 A/m^2^) and the insufficient concentration of Mg^2^⁺ and Ca^2^⁺ ions to undergo precipitation, given the diluted nature of the solution. Magnesium and calcium removal rates reached only 42% and 38%, respectively. Specific energy consumption increased proportionally with current density. The precipitated Mg(OH)_2_ exhibited a gelatinous consistency, resulting in electrical resistance within the cell and at the electrode interface, thereby reducing the calcium removal efficiency. These low removal efficiencies were attributed to the pH range of 4.0 to 7.4 achieved during the tests as the approach considered was to alkalinize the solution to a pH greater than 4, with the objective of employing ion exchange resins [25]. However, the final content of divalent cations was higher than 140 ppm, which was still considered high for the efficient application of ion exchange resins as this technology is typically recommended for trace removal.

The PC1 solution demonstrates a notable removal of Mg and Ca. In most of the tests, the Mg concentration was reduced from 880 ppm to 2 ppm. The highest removals of Mg and Ca were observed in the PC15 and PC12 tests, with final concentrations of 2 ppm and 9 ppm, respectively. For calcium, its concentration was reduced from 700 ppm to 15 ppm only when the final pH was above 12. A particular case was that of the PC13 test, which showed low removal rates of magnesium and calcium. This was the last test performed, and the degradation of the cathode surface (titanium electrode coated with iridium oxide) was observed. Part of the electrode coating detached, increasing the electrical resistance and decreasing the efficiency of the process. In this test, the Mg concentration was only reduced to 462 ppm.

For the LC1 solution, the current density conditions that yielded optimal impurity removal in the PC1 solution—specifically 1000 and 1200 A/m^2^—were selected. Under these conditions, a final pH exceeding 12 was achieved. At a current density of 1000 A/m^2^, the calcium concentration decreased from an initial 1260 ppm to 31 ppm, while the magnesium concentration was reduced from 440 ppm to 2 ppm. Similarly, at 1200 A/m^2^, the final concentrations of calcium and magnesium were 29 ppm and 2 ppm, respectively. These findings underscore the efficiency of the process in impurity removal and solution alkalization.

The initial calcium concentration in the LC1 solution was significantly higher than in the PC1 solution, which accounted for the lower calcium removal in LC1. However, the lithium recovery percentage was lower.

In the P1 solutions (Figure 5), lithium recovery ranged from 91% to 99%. This high recovery was attributed to the highly diluted nature of the solution and the elevated solubility of lithium, which minimized its co-precipitation with magnesium and calcium.

For the concentrated solutions PC1 and LC1, the lithium recovery percentage was higher at a pH above 12. This was because the surface charge of Mg(OH)_2_ remained positive up to this pH [32,35], at which point it became neutral, releasing previously adsorbed ions such as Cl⁻, which may have been associated with Li⁺ ions. The PC12 and PC15 tests, at 1000 and 1200 A/m^2^, respectively, reached a pH of 12 with removal efficiencies exceeding 99% for Mg and 98% for Ca. The specific energy consumption for the removal of both cations was lower in PC12, with a value of 27.33 kWh/kg Li.

The PC1 solution, with a higher Na^+^ concentration than Li^+^, exhibited competition between Li^+^ and Na^+^ for adsorption sites occupied by the Cl⁻ anion due to the high surface adsorption capacity of Mg(OH)_2_. A higher Na^+^ concentration reduces the competition from Li ions [35]. In contrast, in the LC1 solution, lithium concentration predominated over other cations, reducing competition for adsorption by Mg(OH)_2_. However, at a pH of 12, lithium was neutralized and a significant amount was trailed with the hydroxides, resulting in a lower lithium recovery percentage.

In summary, during the electrodialysis process for separating Mg and Ca, two chemical compositions, PC1 and LC1, were compared. In the PC12 and PC15 tests, conducted at current densities of 1000 and 1200 A/m^2^ with a pH above 12, a removal efficiency of 99.8% for Mg and 98% for Ca was achieved, along with high lithium recovery (96–99%) at a higher Na^+^ concentration. The lowest energy consumption was 27.33 kWh/kg Li.

In contrast, in the LC1 brine, a removal efficiency of 99.5% for Mg and 97.7% for Ca was achieved using current densities of 1000 A/m^2^ (LC12) and 1200 A/m^2^ (LC11), with a pH above 12. However, lithium recovery was lower, ranging from 77% to 88%, while the specific energy consumption (SEC) was also lower compared to the PC1 brine with a value of 20.28 kWh/kg Li for the LC12 test.

To optimize the process and reduce energy consumption in electrodialysis, it is suggested for future studies to adjust operational variables such as the concentration and feed flow rate in each compartment.

The brines selected as PC2 and LC2 corresponded to those obtained in the PC12 and LC12 tests, respectively.

### 3.3. Membrane Electrolysis Process Results

Based on the results obtained in Stage 2, the chemical compositions obtained are presented in Table 5. These brines were considered suitable for processing through ion exchange resins to remove trace amounts of Mg and Ca to levels below 1 ppm. The chemical composition of the LC2 brine, with low K content, was considered acceptable to produce Li_2_CO_3_ using Na_2_CO_3_. To assess the feasibility of producing LiOH by membrane electrolysis with these solutions, the selectivity of ion exchange membranes for monovalent ions (Li^+^, K^+^, and Na^+^) was evaluated.

#### 3.3.1. Selectivity and Migration Rate Selectivity of Na^+^, K^+^ and Li^+^

**Figure 6:**
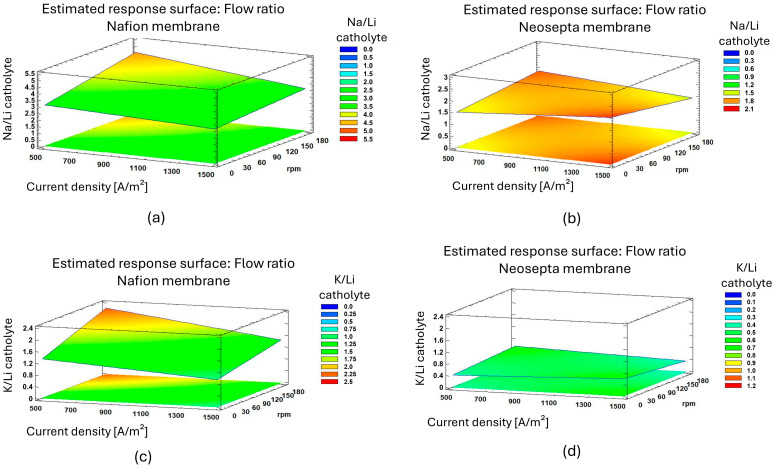
Na^+^ and K^+^ migration rates according to membrane, rpm, and electric current density. (**a**) Na/Li ratio in Nafion 117 membrane; (**b**) Na/Li ratio in Neosepta CMS membrane; (**c**) K/Li ratio in Nafion 117 membrane; (**d**) K/Li ratio in Neosepta CMS membrane.

The migration rate of monovalent ions for the Nafion 117 and Neosepta CMS membranes was studied using the PC2 solution, which had an initial Na/Li concentration ratio of 3.6 and K/Li of 1.26. In Figure 6, the use of Nafion 117 and Neosepta CMS membranes in the cathodic compartment is analyzed to evaluate the influence of these cations on the migration of Li.

In the cathodic compartment, the Na/Li migration rates for the Nafion 117 membrane (Figure 6a) were similar to the ratio of their concentrations, which may indicate that the transport mechanism is primarily controlled by diffusion. When operating at a low current density (500 A/m^2^) and high flow velocities (170 rpm), it was observed that the Na⁺ ion flux was greater compared to the Li⁺ ion flux. This was because the increase in flow velocity favored the convective transport of Na⁺, limiting the migration of lithium, where the minimum value reached was Na/Li = 2.6.

In Figure 6c, the migration rates of Li^+^ and K^+^ for the Nafion 117 membrane are analyzed. At a higher current density of 1500 A/m^2^ and a flow rate of 40 rpm, Li exhibits a migration rate competitive with K^+^. In this case, both ions compete and reach the catholyte in the same proportion. Increasing the flow rate to 170 rpm improves the convection-driven transport of both Li^+^ and K^+^. On the other hand, in most combinations, the transport of K^+^ is higher than that of Li^+^. This is because the lithium ion, having a larger hydration radius than K^+^, requires more energy to dehydrate and pass through the membrane channels, which reduces its transport rate. In contrast, the K⁺ ion with a smaller hydration radius, exhibits greater mobility through the membrane [36]. The higher ionic mobility of K^+^ negatively impacts the transport rate of Li^+^ [31,37]. To enhance Li^+^ transport, increasing the flow rate is necessary, which also benefits the transport of K^+^.

For the Neosepta CMS membrane, Figure 6b shows the migration rate relationship between Na^+^ and Li^+^. When these cations pass to the cathodic side, the difference in migration rates between Li^+^ and Na^+^ is smaller compared to that for the Nafion 117 membrane. On the other hand, in subfigure d, the influence of K^+^ as a coexisting cation on the Li^+^ migration rate in the Neosepta CMS membrane is analyzed. It is observed that the migration rate of Li^+^ is higher than that of K^+^ although the latter slightly increases its migration rate with the increase in current density [38].

#### 3.3.2. Percentage of Recovery and Electric Power Consumption

**Figure 7:**
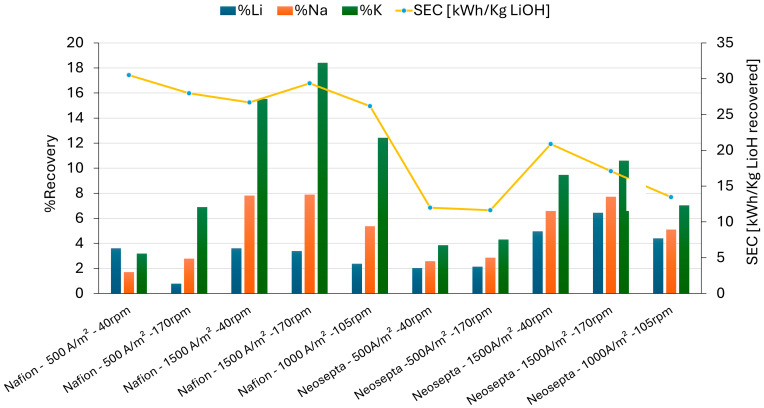
Results of percentage recovery of monovalent cations through Nafion 117 and Neosepta CMS membranes at different current density and rpm values.

Figure 7 presents a summary of the recovery percentages of the various cations, highlighting the differences between the tests using Nafion 117 and Neosepta CMS membranes.

Figure 7 shows a greater influence of current density than rpm on the different recovery percentages in the catholyte. The increase in recirculation speed results in a slight decrease in specific energy consumption and a slight increase in ion transport.

Regarding the membranes, it was observed that ion transport was higher in the Nafion 117 membrane, primarily for the K⁺ ion, to the detriment of Li⁺ transport. The channels of the Nafion 117 membrane were formed by the aggregation of hydrated sulfonate groups, creating suitable pathways for the passage of monovalent and multivalent cations [39]. This allowed ions to pass through the membrane more easily [40].

For the Neosepta CMS membrane, a higher recovery of Li was observed compared to that for the Nafion 117 membrane, along with a lower migration of sodium and potassium. Both membranes had channels formed by fixed sulfonate groups, but the difference lay in their properties. The Nafion 117 and Neosepta CMS membranes exhibited different degrees of cross-linking and surface structures. The Neosepta CMS membrane had an ion exchange capacity of 2.0–2.5 meg/g, a water absorption rate of 26.6%, and a channel diameter of 1–2 nm [37] while the Nafion 117 membrane had a lower ion exchange capacity (0.9 meg/g), lower water absorption (17.4%), and a larger channel diameter (2–5 nm) [39,41]. The Neosepta CMS membrane consists of cross-linked sulfonated co-polymers of vinyl compounds, which are attached to a synthetic reinforcement fabric [37], while the Nafion 117 membrane was a perfluorinated sulfopolymer membrane. Additionally, the Nafion 117 membrane had a greater thickness compared to the Neosepta CMS. Presumably, a greater thickness could have led to an increase in the size of the percolation channels [37], which would explain the higher ion transport in the Nafion 117 membrane despite its lower water content.

On the other hand, the Neosepta CMS membrane, since it had been designed to be selective for monovalent cations, was likely to have a layer of positively charged ionic groups on the membrane surface, as mentioned by Le Xuan et al. [42]. The results showed that the Neosepta CMS membrane demonstrated better selectivity for lithium among monovalent cations compared to the Nafion 117 membrane. The Neosepta CMS membrane had specific characteristics that would allow for a different interaction with the ions. Crossing the solution–membrane interface requires overcoming an energy barrier due to the partial dehydration of the ions. This barrier is greater in membranes selective for monovalent ions as their modification makes the membrane surface more hydrophobic [43]. This is influenced not only by the hydrated radio and membrane channels but also by the ionic radius order of the ions, K^+^ > Na^+^ > Li^+^. In the case of lithium, it has a larger hydrated ionic radius compared to Na^+^ and K^+^ ions [36,44], but upon dehydration, it presents the smallest ionic radius among the alkali metal group. This would allow it to move more easily within the membrane structure by freeing itself from the friction effects of its hydration shell. This could explain the better lithium transport observed for the Neosepta CMS membrane, which would allow the partial dehydration of lithium ions, improving its competitiveness with Na^+^ and K^+^ ions. However, since K^+^ has a larger ionic radius and a smaller charge density, it would have weaker electrostatic interactions with the membrane’s surface layer, facilitating its passage [36]. Therefore, the transport rate of K+ was always the highest.

The Li recovery with the Neosepta CMS membrane exceeded twice that obtained with Nafion 117. The recovery values obtained for Nafion 117 reached a maximum of 3.6% Li, with a specific energy consumption (CEE) of 29.36 kWh/KgLiOH, while for Neosepta CMS, the recovery was 6.44% Li with a CEE of 6.09 kWh/KgLiOH (Figure 7).

**Figure 8:**
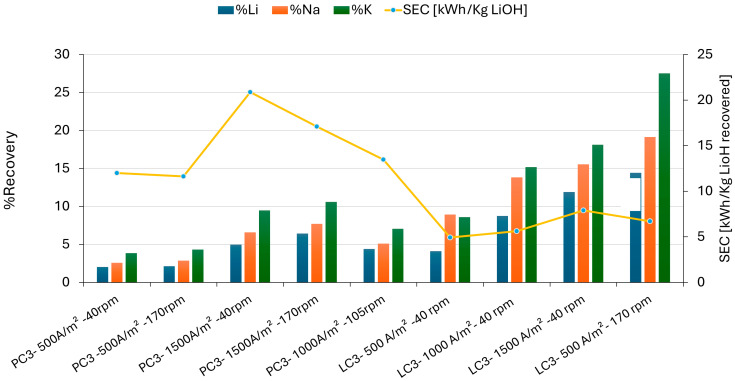
Results of percentage recovery of monovalent cations through Neosepta CMS membranes at different current density and rpm values using LC2 and PC2 brine.

The differences in recovery and energy consumption in the various chemical compositions of the catholyte PC3 and LC3 using the Neosepta CMS membrane can be compared in Figure 8. The Neosepta CMS membrane exhibited higher salt transport and lithium recovery when using the LC2 brine as the anolyte.

Although the initial composition in the anolyte LC2 had a lower impurity content compared to lithium, the migration rate of K^+^ predominated due to its smaller hydrated radius, which provided it with greater mobility within the solution. Through the membrane, a recovery of 27–53% for K^+^ was achieved, compared to only 14.43% for Li, with a specific energy consumption (CEE) of 6.70 kWh/KgLiOH (Test LC3). As shown in Figure 8, lithium recovery was higher at a low current density (500 A/m^2^) and high flow (170 rpm), indicating that the migration of Li was favored by convection transport due to the flow speed applied to the electrolyte. The Neosepta CMS membrane showed better lithium recoveries in the LC3 solution. With an initial concentration of Li and a lower concentration of impurities in LC2 (anolyte), the resulting solution in the catholyte LC3 had 14.44% Li recovered, compared to the 6.4% Li obtained in PC3.

In Stage 3, the effect of each membrane on the lithium migration rate was observed. For the Nafion 117 membrane, the concentration of salts in the solution negatively impacted lithium migration, where an initial Na/Li ratio of 3 favored its transport mainly by diffusion. In the case of the Neosepta CMS membrane, its selectivity for monovalent cations also influenced the lithium migration rate. However, it was not enough to overcome the concentration and migration rate of sodium. For both membranes, increasing the current and flow benefited the migration rate of both lithium and potassium. In each operating condition of the system, the concentration of K^+^ in the catholyte was higher, demonstrating that a higher concentration of Na^+^ does not influence K^+^ migration but it does affect lithium migration. This is due to the order of their hydrated radios (Li^+^ > Na^+^ > K^+^), which influences the migration order. In the LC3 solution, K^+^ recovery still predominated at 27.53% despite the much lower concentration of K^+^, meaning that no effect from the concentration, current, or flow rate was detected in reducing the migration rate and recovery of K^+^.

#### 3.3.3. Statistical Analysis Results

The results of the analysis of main effects and interactions for evaluating the selectivity of the Na^+^ and K^+^ ions are presented in Table 6. Regarding the analysis results, the average Na/Li ratio was 7.6522, with a standard error of 0.7882. The membrane type (C) had the greatest absolute impact on the Na/Li ratio, with a significant negative effect of −3.9917 when switching to Neosepta CMS membrane, followed by the flow rate (B), with a positive effect of 3.5183, while current density (A) showed a more modest positive effect of 0.5086. Among the interactions, the most relevant was the BC interaction (flow rate and membrane type), with a negative effect of −3.6652. The AB (−3.4307) and AC (−0.6689) interactions had smaller effects. This shows that the membrane type is the most important factor in controlling the Na/Li ratio. The independence between factors was confirmed by VIF values, all of which were equal to 1.0.

As per the results analysis for K/Li, the average K/Li ratio was 4.9978, with a standard error of 0.6153. The membrane type (C) was again the most influential factor, with a significant negative effect of −4.6004 when switching to the Neosepta CMS membrane, followed by the flow rate (B), with a positive effect of 3.2652, while current density (A) showed a nonsignificant effect of −0.0651. The BC interaction stood out as relevant, with a negative effect of −3.3746, while the AB interaction showed a negative effect of −2.6120. On the other hand, current density (A) and its interaction with membrane type (C) were not significant.

The analysis of variance (ANOVA) supported the results obtained for the K/Li ratio, identifying the membrane type as the only factor with a significant effect (*p* = 0.0334, F = 13.98). Flow rate (B) had a trend towards significance (*p* = 0.0982) while current density (A) and its interactions did not reach statistical significance, which was consistent with what was reported by Saracco [45] for the Neosepta CMS membrane. The model explained 90.7% of the variability (R^2^). On the other hand, the analysis of variance (ANOVA) for the Na/Li ratio indicated that none of the factors had a significant effect (*p* > 0.05). Membrane type (C) had the greatest relative impact (F-Ratio = 6.41, *p* = 0.0853), followed by current density (B) (F-Ratio = 3.98, *p* = 0.1398). The model explained 86.2% of the variability (R^2^), with an adjusted R^2^ of 58.6%. Overall, membrane type appeared to be the most relevant factor for future investigations.

### 3.4. Challenges and Future Prospects

The development of an efficient and sustainable process for direct lithium extraction faces several challenges that must be addressed in order to enhance its industrial application. In this study, electrochemical processes were explored as an ecological alternative, avoiding the use of chemical reagents. The results obtained are promising in terms of impurity removal. However, key obstacles remain in optimizing energy efficiency. From Table 7, it can be inferred that further optimization is required to minimize energy costs, which could be achieved by refining operational conditions. Future efforts should focus on improving concentration processes, using clean thermal energy or integrating conventional electrodialysis or nanofiltration instead of thermal evaporation. The biggest challenge for DLE processes is to obtain lithium-rich solutions with higher concentrations than those currently achieved.

The electrochemical removal rates of Mg and Ca reached 99.8% and 98% in the concentrated solutions PC1 and LC1, which presented Mg/Li and Ca/Li ratios of 0.084 and 0.066, respectively. Similar removals were achieved through chemical precipitation [26]. However, the difficulty in removing calcium increased the energy consumption required to 27.33 kWh per kg of lithium. Similarly, Nieto et al. [32] used electrochemical alkalinization to reduce divalent cations to below 2 ppm from natural brine, with a minimum energy consumption of 62 kWh per cubic meter of brine containing 0.13% lithium by mass (equivalent to approximately 43 kWh per kg of lithium). This stage generates Mg(OH)_2_ and Ca(OH)_2_ solids as byproducts. The former has various applications across different industrial sectors, such as the steel, food, and pharmaceutical industries [46], while the latter holds potential for use in thermochemical heat storage systems [47,48]. To achieve this, separation and refinement processes for these compounds are required [49].

An alternative is to alkalinize the brine to a pH greater than 4 so that the process can be integrated with ion exchange resins to remove Mg^2+^ and Ca^2+^ [25,26]. However, it was determined that at a pH between 4 and 7, the removal was less than 42%. The challenge lies in finding the balance between the degree of alkalinization and the remaining Mg and Ca content, which would allow for reduced costs in capture and desorption cycles in ion exchange columns.

The difference in selectivity between the Nafion 117 and Neosepta CMS membranes was determined, highlighting the need to improve selectivity toward lithium in addition to enhancing the purity of the initial LiCl anolyte. The challenge of removing impurities from lithium-rich brines remains a significant barrier to producing high-purity lithium compounds, particularly for battery materials. The results of this study show that the high rates of magnesium and calcium removal, along with high lithium recovery, make it feasible to use the PC12 brine to achieve suitable quality for lithium carbonate production via carbonation with Na_2_CO_3_ as the sodium content does not pose a problem for Li_2_CO_3_ precipitation. However, the impurities of sodium and potassium make difficult the production of LiOH via membrane electrolysis and subsequently obtain LiOH·H_2_O crystals efficiently. It was demonstrated that Na^+^ and K^+^ negatively affect lithium transport in membranes, which was consistent with studies by Chen et al. [31], which results in an undesired energy consumption in the membrane electrolysis process while the presence of Na^+^ and K^+^ in the final catholyte does not prevent the crystallization and formation of LiOH H_2_O because aqueous LiOH has lower solubility than NaOH and KOH [50]. Future research should focus on selective removal techniques, such as nanofiltration, that can improve the overall quality of the final lithium solution. A comparative analysis will be required to determine the optimal balance between the energy demand of this approach and the additional energy required for the prior removal of Na^+^ and K^+^, ensuring the most efficient and feasible solution.

Regarding the chemical composition of the complex lithium solution used in this work, it is essential to address the challenges associated with real solutions derived from direct lithium extraction (DLE). The authors consider the initial solutions P1 and L1 as representative for the concentration stage (Stage 1) while variations may occur in Stages 2 and 3 when using real solutions from DLE or natural brines. In general, brines exhibit a wide range of ions beyond those considered in this study, such as Fe, Zn, Mn, and others [19], which may also be present in solutions derived from DLE and subsequently in each post-treatment stage. In this study, the analysis focused on impurities like Na^+^, K^+^, Ca^2+^, and Mg^2+^, which are commonly carried over in direct lithium extraction processes, especially those based on adsorption and ion exchange [18]. Some challenges associated with feeding real brines include the presence of organic matter or other compounds such as Fe^2^⁺, as seen in solutions obtained from the recovery of spent-Li batteries [51]. These compounds may interfere with adsorption processes and membrane performance, ultimately affecting lithium recovery. Elements like sulfate and iron may form insoluble compounds, impacting the purity of Li_2_CO_3_. However, the concentrations of these impurities after DLE are relatively low, suggesting that their effects could be considered negligible in real scenarios [18,19].

An aspect to optimize in the future is the effect of temperature. On one hand, increasing the temperature will contribute to enhancing ionic mobility and its interaction with the functional groups in the membranes, which would influence the migration rate and membrane selectivity for monovalent ions other than lithium. In the production of LiOH via membrane electrolysis, better performance has been reported when operating at temperatures between 70 and 80 °C [13]. Therefore, investigating how temperature affects membrane selectivity is an interesting approach to optimizing the process. Regarding electrochemical alkalinization, the process has been conducted at room temperature to avoid increasing energy consumption. Additionally, higher temperatures will increase the solubility of Mg(OH)_2_ and Ca(OH)_2_, which would likely reduce removal efficiency. However, it is worth exploring their effect on ionic mobility and the particle size of Mg(OH)_2_ and Ca(OH)_2_ obtained. Similarly, the generation of H_2_ in the cathodic half-reaction could become a potential energy source for thermal management of the process. These considerations could be addressed in future studies.

## 4. Conclusions

The present study developed a pre-treatment process for the concentration and separation of impurities from a complex LiCl solution obtained from an EDL process. The objectives, both general and specific, were addressed in stages through a series of experimental tests and analyses, with the following conclusions.

Stage 1: The concentration process by the evaporation of complex LiCl solutions containing impurities, mainly Na, K, Ca, and Mg, was determined and applied. A LiCl concentration of 1% Li was achieved through constant evaporation without reaching saturation. This process can be improved using alternative techniques to prevent water losses such as reverse osmosis or conventional electrodialysis.

Stage 2: The proposed electrodialysis process for the alkalinization and removal of Mg and Ca showed results where more than 99% removal was achieved. Better results were obtained in concentrated solutions (PC1 and LC1) compared to those directly from the EDL process (P1). The best Mg and Ca removals were observed in the tests of PC15 and PC12, achieving 99.8% and 99% Mg removal rates, respectively. For Ca, effective removal was achieved up to a concentration of 15 ppm (98% removal) when the final pH was greater than 12. These results indicated energy consumptions of 32.4 and 27.33 kWh/kg of Li treated in solution, with lithium recovery ranging from 96% to 99%. In solution LC1, 97% Ca removal (29 ppm Ca) and 99.55% Mg removal (2 ppm) were achieved, with energy consumptions of 24.91 and 20.28 kWh/Kg Li, respectively. Lithium recovery ranged from 77% to 81%.

Stage 3: The membrane electrolysis process was used to assess how the migration rates of these ions influence lithium recovery. Two commercial cationic membranes were used: a cationic Nafion 117 and a monovalent-selective Neosepta CMS. It was observed that the type of membrane impacted the ion migration rates under operating conditions of 500–1500 A/m^2^ and 40–170 rpm. The membrane type influenced the preference for lithium migration rates. Monovalent-selective membranes, which are usually designed for multivalent cations, influence monovalent cations due to their structural properties and physico-chemical characteristics.

The results showed that the final concentration in the catholyte followed the order K^+^ > Na^+^ > Li^+^. A higher lithium recovery and lower energy consumption were observed using the Neosepta CMS membrane, achieving the highest lithium recovery at 14.43%. The specific energy consumption was 6.09 kWh/Kg LiOH for solution PC3 and 6.70 kWh/Kg LiOH for solution LC3.

## Figures and Tables

**Table 1:** Chemical compositions of LiCl solutions.

Solution	pH	%Li	%Na	%K	%Mg	%Ca	%LiCl	Na/Li	K/Li	Mg/Li	Ca/Li
P1	1.2	0.076	0.318	0.095	0.007	0.006	0.47	4.184	1.25	0.095	0.075
L1	1.3	0.055	0.002	0.004	0.002	0.006	0.34	0.04	0.074	0.038	0.109

**Table 2:** Current density applied in electrodialysis for alkalinization (A/m^2^).

Experiment Number	1	2	3	4	5
P1	77	77	113	153	153
PC1	1000	1000	1100	1200	1200
LC1	1000	1000	1100	1200	1200

**Table 3:** Experimental tests for membrane electrolysis solution PC2—Stage 3.

N° Exp	Solution	Current Density (A/m^2^)	rpm	Membrane
1	PC2	500	40	Nafion 117
2	PC2	1500	40	Nafion 117
3	PC2	500	170	Nafion 117
4	PC2	1500	170	Nafion 117
5	PC2	1000	105	Nafion 117
6	PC2	500	40	Neosepta CMS
7	PC2	1500	40	Neosepta CMS
8	PC2	500	170	Neosepta CMS
9	PC2	1500	170	Neosepta CMS
10	PC2	1000	105	Neosepta CMS

**Table 4:** Chemical compositions of the solutions obtained after evaporation.

	%Li	%Na	%K	%Mg	%Ca
PC1	1.050	3.820	1.320	0.088	0.070
LC1	1.144	0.047	0.085	0.044	0.126

**Table 5:** Chemical compositions of the solutions PC2 and LC2.

Solution	pH	%Li	%Na	%K	%LiCl	Na/Li	K/Li
PC2	12.5	1.05	3.82	1.32	6.41	3.64	1.26
LC2	12	1.14	0.05	0.09	6.99	0.04	0.07

**Table 6:** Results of the main effects and interaction analysis for Na/Li and K/Li ratios.

	Results for Ratio Na/Li			Results for Ratio K/Li		
Effect	Estimate	Stnd. Error	V.I.F.	Estimate	Stnd. Error	V.I.F.
Average ratio Na/Li	7.65222	0.788233		4.99781	0.615292	
A: Current Density	0.508602	1.76254	1.0	−0.0650875	1.37584	1.0
B: Flow	3.5183	1.76254	1.0	3.26524	1.37584	1.0
C: Type of Membrane	−3.99172	1.57647	1.0	−4.60042	1.23058	1.0
AB	−3.43075	1.76254	1.0	−2.61201	1.37584	1.0
AC	−0.668897	1.76254	1.0	−0.196738	1.37584	1.0
BC	−3.6652	1.76254	1.0	−3.37461	1.37584	1.0

**Table 7:** Specific energy consumption in the preliminary process developed.

Initial Solution	Unit	Evaporation	Alkalinization (Electrodialysis)	Membrane Electrolysis
L1	kWh/kg Li	52.45 (LC1)	20.28 (LC12)	22.21 (LC3)
P1	kWh/kg Li	52.38 (PC1)	27.33 (PC12)	33.46 (PC3)

## Data Availability

The original contributions presented in this study are included in the article. Further inquiries can be directed to the corresponding author.
